# Machine learning algorithm for predict the in-hospital mortality in critically ill patients with congestive heart failure combined with chronic kidney disease

**DOI:** 10.1080/0886022X.2024.2315298

**Published:** 2024-02-15

**Authors:** Xunliang Li, Zhijuan Wang, Wenman Zhao, Rui Shi, Yuyu Zhu, Haifeng Pan, Deguang Wang

**Affiliations:** a Department of Nephrology, The Second Affiliated Hospital of Anhui Medical University, Hefei, China; b Institute of Kidney Disease, Inflammation and Immunity Mediated Diseases, The Second Affiliated Hospital of Anhui Medical University, Hefei, China; c Department of Epidemiology and Biostatistics, School of Public Health, Anhui Medical University, Hefei, China; d Inflammation and Immune Mediated Diseases Laboratory of Anhui Province, Hefei, China

**Keywords:** Congestive heart failure, chronic kidney disease, machine learning, mortality, critically care

## Abstract

**Background:**

The objective of this study was to develop and validate a machine learning (ML) model for predict in-hospital mortality among critically ill patients with congestive heart failure (CHF) combined with chronic kidney disease (CKD).

**Methods:**

After employing least absolute shrinkage and selection operator regression for feature selection, six distinct methodologies were employed in the construction of the model. The selection of the optimal model was based on the area under the curve (AUC). Furthermore, the interpretation of the chosen model was facilitated through the utilization of SHapley Additive exPlanation (SHAP) values and the Local Interpretable Model-Agnostic Explanations (LIME) algorithm.

**Results:**

This study collected data and enrolled 5041 patients on CHF combined with CKD from 2008 to 2019, utilizing the Medical Information Mart for Intensive Care Unit. After selection, 22 of the 47 variables collected post-intensive care unit admission were identified as mortality-associated and subsequently utilized in the development of ML models. Among the six models generated, the eXtreme Gradient Boosting (XGBoost) model demonstrated the highest AUC at 0.837. Notably, the SHAP values highlighted the sequential organ failure assessment score, age, simplified acute physiology score II, and urine output as the four most influential variables in the XGBoost model. In addition, the LIME algorithm explains the individualized predictions.

**Conclusions:**

In conclusion, our study accomplished the successful development and validation of ML models for predicting in-hospital mortality in critically ill patients with CHF combined with CKD. Notably, the XGBoost model emerged as the most efficacious among all the ML models employed.

## Background

Congestive heart failure (CHF) persists as a prominent contributor to morbidity and mortality worldwide, affecting over 23 million individuals [1]. Concurrently, chronic kidney disease (CKD) is prevalent in CHF patients and is associated with an unfavorable prognosis in terms of global and cardiovascular mortality prognosis [2]. According to pivotal CHF trials, the prevalence of CKD ranges from 32 to 50% [3]. The prognosis for patients with CHF combined with CKD is notably grim, exacerbating as renal function deteriorates, ultimately leading to elevated mortality rates [4]. Recent research underscores the importance of early identification of critically ill individuals at risk of rapid deterioration, with potential implications for improved clinical outcomes [5]. Predictive models tailored to identify high-risk patients with CHF combined with CKD for in-hospital mortality offer a promising avenue for healthcare professionals to allocate resources more efficiently. This facilitates personalized interventions and intensified monitoring for those individuals most likely to benefit. Therefore, the development of accurate prediction models capable of reliably estimating an individual’s survival prognosis holds significant potential for advancing therapeutic practice.

In leveraging substantial datasets, encompassing demographics, diagnoses, regularly measured values, and treatments from electronic health records, machine learning (ML) algorithms presents a promising avenue to mitigate mortality rates in critically ill patients with CHF combined with CKD. These sophisticated, data-driven strategies excel in handling high-dimensional data, model intricate relationships, and identifying vital predictors linked to outcomes. A growing body of evidence demonstrates that ML techniques outperform traditional models [6, 7]. ML approaches have gained prominence in disease prognostication, allowing clinicians, with well-constructed prediction models, to identify patients at high risk for poor outcomes, facilitating more timely interventions and yielding improved results [8–10]. Notably, analyses of outcome prediction in patients with CHF combined with CKD are relatively scarce. Therefore, the objective of this research is to forecast in-hospital mortality rates among critically ill patients with CHF combined with CKD using the ML method.

## Methods

### Database introduction

The Medical Information Mart for Intensive Care IV (MIMIC IV) database stands as a thorough, de-identified clinical dataset, sanctioned by both the Beth Israel Deaconess Medical Center and the Massachusetts Institute of Technology [11]. The necessity for individual patient consent and ethically informed consent declarations was waived, given that the study had no impact on clinical decision-making, and the anonymity of all patients in the database was maintained [12]. The author XL successfully completed the protection of human research participants exam and secured a certificate authorizing access to the database (No. 35970146).

### Study population

All patients within the MIMIC IV database diagnosed with CKD combined with CHF were included in this study. The diagnosis of CKD and CHF was relied on the International Classification of Diseases, Ninth Revision (ICD-9) and International Classification of Diseases, Tenth Revision (ICD-10) codes documented by hospital personnel during patient discharge (Supplementary Table S1). Only the first admission will be considered for patients with a history of multiple ICU admissions. Exclusions comprised patients below 18 years old and those with an ICU stay of less than 24 h.

### Data collection

We used Navicat Premium software for data extraction from the MIMIC IV database. Taking into account all available parameters and utilizing clinical expertise, we selected 47 candidate variables based on association with outcomes. We collected age, sex, weight, and ethnicity as demographic information for this study. Comorbidities included cerebrovascular disease, rheumatic disease, chronic obstructive pulmonary disease (COPD), diabetes, peripheral vascular disease, myocardial infarction, peptic ulcer disease, liver disease, paraplegia, cancer, and acute kidney injury. The patient’s CKD stage was also collected. We gathered initial values of vital sign data, including temperature, respiration rate, mean arterial pressure, heart rate, systolic blood pressure, and oxygen saturation, within 24 h of admission. For biochemical indices, we collected initial values within the first 24 h after admission for serum sodium, serum potassium, bicarbonate, serum chloride, serum calcium, serum glucose, serum creatinine, international normalized ratio, anion gap, blood urea nitrogen, white blood cell, platelets, hemoglobin, hematocrit, prothrombin time, and partial thromboplastin time. Blood urea nitrogen is a blood test that measures the level of urea nitrogen in the bloodstream. It is commonly used to assess kidney function. Elevated BUN levels can indicate kidney dysfunction or other medical conditions. Prothrombin time is a laboratory test that measures how long it takes for blood to clot. It is often used to assess the function of the coagulation (blood clotting) system and to monitor the effects of anticoagulant medications. We recorded the total amount of urine voided within the initial 24 h following admission to the ICU. Within the same time frame, we recorded medical treatments such as mechanical ventilation, vasopressors, and renal replacement therapy. In the initial 24 h post-admission, we assessed the first values of the sequential organ failure assessment (SOFA) score and the simplified acute physiology score II (SAPS II) as severity scores of illness. The SOFA score is a clinical tool used to assess the severity of organ dysfunction/failure in critically ill patients. It evaluates the function of six organ systems: neurological, renal, coagulation, hepatic, cardiovascular, and respiratory. SAPS II is a severity-of-illness scoring system used to predict the risk of mortality in critically ill patients. It takes into account several physiological parameters, age, and underlying comorbidities to estimate the probability of survival. The decision to use SOFA and SAPS II scores was based on their wide recognition and established utility in assessing disease severity in critically ill patients across various studies and clinical settings. These scoring systems offer a comprehensive evaluation of organ dysfunction and physiological derangements, allowing for a reliable quantification of disease severity.

### Preprocessing of data

Missing values are common in the MIMIC IV database, and all variables in this study had missing values of less than 20% (Supplementary Table S2). We used multiple interpolation methods to fill in the missing data. The least absolute shrinkage and selection operator (LASSO) regression can construct a penalty function to obtain a finer model, which is a data downscaling algorithm that helps to filter out key factors affecting the results, improve model performance, and reduce overfitting. Therefore, we used LASSO regression to identify variables that may be associated with mortality. For the LASSO analysis, we utilized the entire dataset for model development and implemented cross-validation to optimize the tuning parameter (λ). To enhance the robustness of our model, we adopted a 10-fold cross-validation strategy, wherein the dataset was partitioned into 10 subsets. The LASSO analysis was then iteratively applied on each fold, with the λ parameter selected based on the minimization of cross-validated error. This approach ensures a comprehensive exploration of the regularization parameter space, ultimately leading to the identification of the optimal λ that maximizes the model’s predictive performance.

### Statistical analysis

Continuous variables in this study were presented as the median and interquartile range (IQR), and the Mann-Whitney test was employed to discern differences between groups owing to their non-normal distribution. Categorical variables were conveyed as numbers and percentages, with group comparisons conducted using either the chi-square test or Fisher’s exact test, as appropriate.

In our analysis, we conducted the statistical analysis using a combination of Python (Version 3.9.12) and R software (Release 4.2.1, Foundation R for Statistical Computing). We utilized several Python and R software packages for data processing; Python software packages include pandas, NumPy, scikit-learn, XGBoost, SHapley Additive exPlanation (SHAP), and, Local Interpretable Model-Agnostic Explanations (LIME) and R software packages include glmnet and ROCR. Statistical significance was defined as a *P* value below 0.05.

### Machine learning

All patients participating in this study were randomly divided into a training set (70%) and a validation set (30%). Six ML techniques: extreme gradient boosting (XGBoost), k-nearest neighbor (KNN), support vector machine (SVM), random forest (RF), decision tree and logistic regression were used to build and validate the model for in-hospital mortality risk. We calculated the accuracy, sensitivity, specificity, area under the curve (AUC), recall, precision, F1 Score, and Matthews correlation coefficient (MCC) of the models for evaluating the predictive performance of different ML models. The testing AUC values corresponding to the different models were compared using paired Delong’s test. The calibration curve is plotted and used to compare the actual with the predicted mortality risk. Based on the AUC, our final candidate model was selected. Since SOFA and SAPS II scores are used as common tools to predict severity and prognosis in critically ill patients, we compared the predictive power of the final model with that of traditional scoring systems. The American Heart Association Get With The Guidelines-Heart Failure (GWTG-HF) risk score is a widely accepted in-hospital mortality risk stratification scoring system. This scoring system is calculated based on patient-related data, including age, systolic blood pressure, blood urea nitrogen, heart rate, serum sodium, COPD, and non-African American ethnicity. We also compared our final model to the GWTG-HF risk score. The SHAP value is a concept rooted in cooperative game theory. It is used to attribute a value to each feature in a prediction, indicating its contribution to the prediction outcome. In the context of ML, SHAP values provide a way to explain the output of a model by quantifying the impact of each feature on that output. We utilized the SHAP values to examine the significance of individual features affecting the model’s output and to depict the relevant features influencing the mortality risks. LIME is a method designed to provide local explanations for the predictions of complex ML models. It aims to explain the predictions of any black-box model by training a simpler, interpretable model on a local subset of the data around the instance being explained. By generating and analyzing a dataset of perturbed instances, LIME facilitates the creation of local models that mimic the intricate model’s behavior around specific cases. This study applies the LIME algorithm to fit the predictive behavior of the model to individuals. Finally, subgroup analyses were performed according to the presence of sepsis, diabetes, paraplegia, cancer, AKI and different CKD stages.

## Results

### Participants

**Figure 1.**
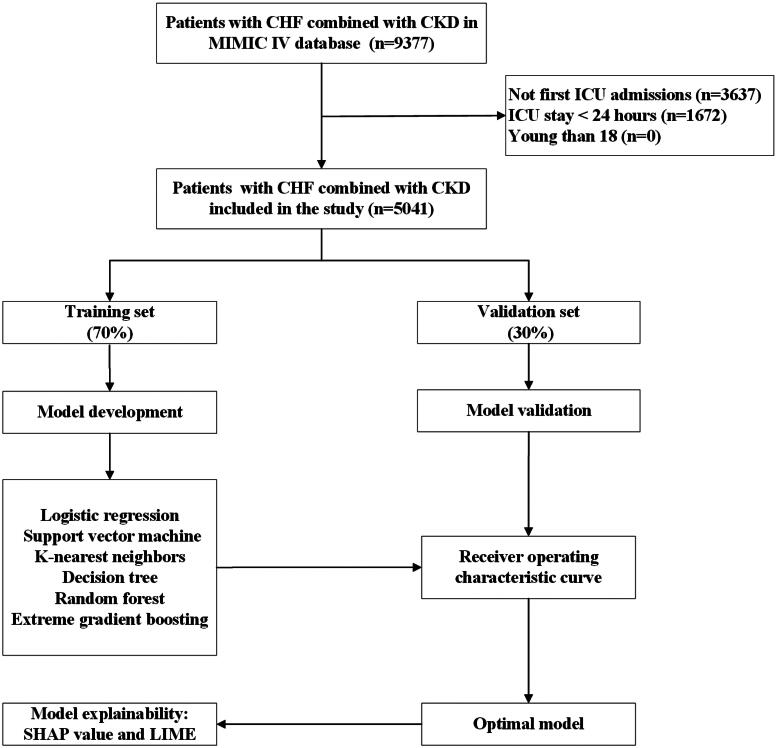
The flowchart of patient selection. Abbreviations: CHF: congestive heart failure, CKD: chronic kidney disease, MIMIC IV: Medical Information Mart for Intensive Care IV, ICU: intensive care unit.

A total of 9377 participants with CHF combined with CKD were determined to be eligible; of these 9377 patients, 3637 were disqualified for non-first ICU admissions, and 1672 patients were excluded due to a length of stay in the ICU of less than 24 h. Finally, 5041 patients met this study’s inclusion and exclusion criteria (Figure 1). The in-hospital death rate among ICU-admitted CHF combined with CKD patients was 18.5% (933/5041). Of these patients, 60.5% (3049/5041) were male, with a median age of 76.9 (IQR: 67.9-84.8) years. Diabetes (2742/5041, 54.4%), sepsis (2095/5041, 41.6%), and COPD (1874/5041, 37.2%) were the top three comorbidities. The demographics, comorbidities, vital signs, biochemical indices, urine output, medical treatments, and severity scores of illness of the patients are listed in Table 1.

**Table 1.** Demographic and clinical characteristics at baseline.

Variables	Total (*n* =5041)	Survivors (*n* =4108)	Nonsurvivors (*n* =933)	*P* value
**Demographics**				
Sex, male, *n* (%)	3049 (60.5)	2485 (60.5)	564 (60.5)	1.000
Age (years)	76.9 [67.9, 84.8]	76.3 [67.0, 84.4]	79.5 [71.4, 86.2]	<0.001
Weight (kg)	79.7 [67.0, 94.5]	80.0 [67.8, 95.3]	76.0 [65.0, 92.0]	<0.001
Ethnicity, *n* (%)				<0.001
White	3384 (67.1)	2783 (67.7)	601 (64.4)	
Black	717 (14.2)	604 (14.7)	113 (12.1)	
Others	940 (18.6)	721 (17.6)	219 (23.5)	
**CKD stage, *n* (%)**				<0.001
Stage 1	62 (1.2)	52 (1.3)	10 (1.1)	
Stage 2	438 (8.7)	387 (9.4)	51 (5.5)	
Stage 3	2092 (41.5)	1776 (43.2)	316 (33.9)	
Stage 4	1384 (27.5)	1051 (25.6)	333 (35.7)	
Stage 5	734 (14.6)	577 (14.0)	157 (16.8)	
Dialysis	331 (6.6)	265 (6.5)	66 (7.1)	
**Comorbidities, *n* (%)**				
Myocardial infarction	1836 (36.4)	1474 (35.9)	362 (38.8)	0.102
Peripheral vascular disease	1115 (22.1)	903 (22.0)	212 (22.7)	0.654
Cerebrovascular disease	661 (13.1)	496 (12.1)	165 (17.7)	<0.001
Dementia	260 (5.2)	203 (4.9)	57 (6.1)	0.169
COPD	1874 (37.2)	1520 (37.0)	354 (37.9)	0.617
Rheumatic disease	229 (4.5)	193 (4.7)	36 (3.9)	0.306
Peptic ulcer disease	169 (3.4)	128 (3.1)	41 (4.4)	0.063
Liver disease	514 (10.2)	370 (9.0)	144 (15.4)	<0.001
Diabetes	2742 (54.4)	2259 (55.0)	483 (51.8)	0.081
Paraplegia	149 (3.0)	98 (2.4)	51 (5.5)	<0.001
Cancer	570 (11.3)	415 (10.1)	155 (16.6)	<0.001
Aids	22 (0.4)	20 (0.5)	2 (0.2)	0.387
Sepsis	2095 (41.6)	1594 (38.8)	501 (53.7)	<0.001
AKI	3259 (64.6)	2515 (61.2)	744 (79.7)	<0.001
**Vital signs**				
Heart rate (beats/minute)	84.0 [72.0, 97.0]	83.0 [72.0, 96.0]	88.0 [75.0, 104.0]	<0.001
MAP (mmHg)	78.0 [68.0, 91.0]	79.0 [69.0, 92.0]	75.0 [66.0, 88.0]	<0.001
SBP (mmHg)	120.0 [103.0, 139.0]	122.0 [105.0, 142.0]	113.0 [99.0, 132.0]	<0.001
Respiratory rate (beats/minute)	19.0 [16.0, 23.0]	19.0 [16.0, 23.0]	21.0 [17.0, 25.0]	<0.001
Body temperature (°C)	36.6 [36.3, 36.9]	36.7 [36.4, 36.9]	36.6 [36.3, 36.9]	<0.001
SpO_2_ (%)	98 [95, 100]	98 [95, 100]	97 [94, 100]	0.013
**Biochemical indices**				
Hematocrit (%)	30.8 [26.7, 35.6]	30.7 [26.6, 35.6]	31.0 [27.1, 36.0]	0.080
Hemoglobin (g/dL)	9.80 [8.50, 11.40]	9.80 [8.50, 11.50]	9.80 [8.40, 11.30]	0.691
Platelets (K/uL)	193 [141, 258]	194 [143, 258]	186 [130, 259]	0.040
WBC (K/uL)	10.20 [7.40, 14.20]	9.90 [7.30, 13.72]	11.60 [8.20, 16.30]	<0.001
Anion gap (mEq/L)	16.0 [14.0, 19.0]	16.0 [13.0, 19.0]	18.0 [15.0, 21.0]	<0.001
Bicarbonate (mmol/L)	23.0 [20.0, 26.0]	23.0 [20.0, 26.0]	22.0 [18.0, 26.0]	<0.001
BUN (mg/dL)	41.0 [28.0, 62.0]	40.0 [27.0, 60.0]	49.0 [33.0, 72.0]	<0.001
Serum calcium (mg/dL)	8.60 [8.10, 9.10]	8.60 [8.10, 9.10]	8.50 [8.00, 9.10]	0.021
Serum chloride (mEq/l)	101 [96, 106]	101 [97, 106]	101 [95, 106]	0.006
Serum creatinine (mg/dL)	2.00 [1.40, 3.20]	1.90 [1.40, 3.10]	2.30 [1.60, 3.50]	<0.001
Serum glucose (mg/dL)	136 [107, 183]	135 [107, 180]	142 [110, 194]	0.006
Serum sodium (mEq/L)	138 [135, 141]	138 [135, 141]	138 [135, 141]	0.930
Serum potassium (mEq/L)	4.40 [4.00, 5.00]	4.40 [4.00, 5.00]	4.50 [4.00, 5.10]	0.046
INR	1.30 [1.20, 1.80]	1.30 [1.10, 1.70]	1.50 [1.20, 2.20]	<0.001
PT (s)	14.7 [12.7, 19.4]	14.5 [12.6, 18.5]	16.3 [13.4, 23.0]	<0.001
PTT (s)	32.6 [28.2, 41.1]	32.1 [28.0, 40.2]	35.3 [29.7, 46.6]	<0.001
**Urine output (mL)**	1270 [635, 2145]	1380 [760, 2271]	740 [256, 1465]	<0.001
**Medical treatments, *n* (%)**				
RRT	695 (13.8)	514 (12.5)	181 (19.4)	<0.001
Vasopressors use	307 (6.1)	178 (4.3)	129 (13.8)	<0.001
Mechanical ventilation	4259 (84.5)	3423 (83.3)	836 (89.6)	<0.001
**Severity scores of illness**				
SOFA score	6.00 [4.00, 9.00]	6.00 [4.00, 8.00]	9.00 [7.00, 12.00]	<0.001
SAPS II	42.0 [35.0, 51.0]	40.0 [34.0, 48.0]	51.0 [42.0, 61.0]	<0.001

Abbreviations: CKD: chronic kidney disease, Aids: acquired immune deficiency syndrome, AKI: acute kidney injury, COPD: chronic obstructive pulmonary disease, MAP: mean arterial pressure, SBP: systolic blood pressure, SpO_2_: oxygen saturation, WBC: white blood cell, BUN: blood urea nitrogen, INR: international normalized ratio, PT: prothrombin time, PTT: partial thromboplastin time, RRT: renal replacement therapy, SOFA: sequential organ failure assessment, SAPS II: simplified acute physiology score II.

### Predictor selection

A total of 47 clinical variables were included in the LASSO regression, and Supplementary Figure S1A shows a plot of the regression coefficients for the model. Each curve represents one variable. At each different input, factors with nonzero coefficients and corresponding nonzero coefficients formed a LASSO model. The LASSO feature selection process is shown in Supplementary Figure S1B. We chose 10-fold cross-validation to further determine the optimal model. The cross-validation error of the model is minimized when λ = 0.0077. Ultimately, 22 variables were still significant predictors of death (Supplementary Table S3). Correlation coefficients between these variables are shown in Supplementary Table S4.

### Model development and validation

**Figure 2.**
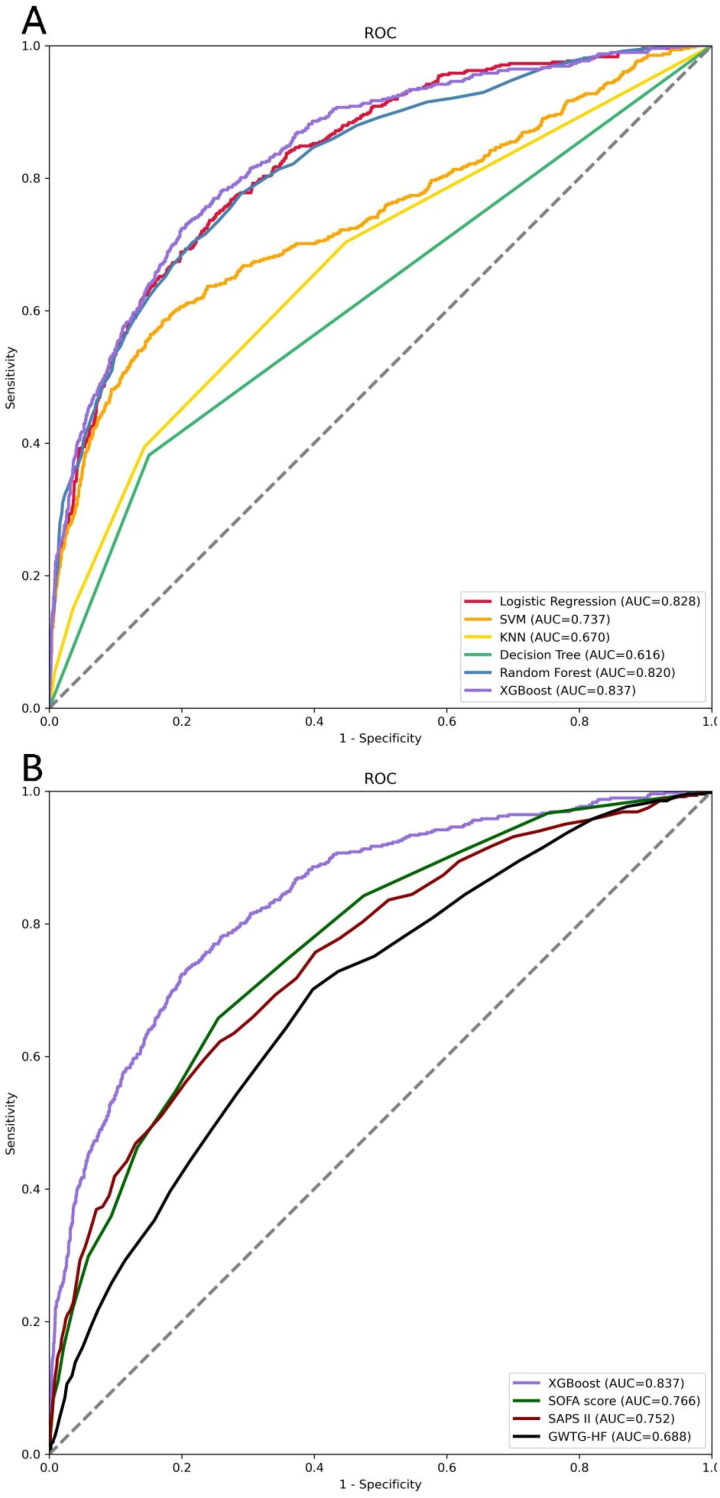
ROC Curves for predicting the incidence of in-hospital mortality with ML models and the traditional severity of illness scores. A ROC curves of six ML models for predicting in-hospital mortality; B ROC curves for the traditional severity of illness scores predicting in-hospital mortality. Abbreviations: ROC: receiver operating characteristic, SVM: support vector machine, KNN, k-nearest neighbors, AUC: area under the curve, SOFA: sequential organ failure assessment, SAPS II: simplified acute physiology score II.

The included patients were randomized into the training set (3528, 70%) and the validation set (1513, 30%), and no significant differences were observed in the variables between the two sets (Supplementary Table S5). We built six ML models (XGBoost, KNN, SVM, RF, decision tree and logistic regression) with 22 variables chosen by LASSO regression as input components. The XGBoost model has the highest AUC (0.837) in the validation set (logistic regression: 0.828; SVM: 0.737; KNN: 0.670; decision tree: 0.616; RF: 0.820) (Figure 2(A) and Supplementary Table S6). Supplementary Table S7 shows the AUC of the six models in the training set. Similarly, the XGBoost model outperformed the SAPS II (AUC: 0.752), SOFA (AUC: 0.766) score and GWTG-HF (AUC: 0.688) (Figure 2(B)). Table 2 displays the results of an evaluation of the AUC, accuracy, sensitivity, specificity, recall, precision, F1 Score, and MCC of these six ML models. Calibration plots for the six ML models are shown in Supplementary Figure S2.

**Table 2.** Performance comparison of the six models in the testing set.

Models	Accuracy	AUC (95% CI)	Sensitivity	Specificity	Recall	Precision	F1 Score	MCC
Logistic regression	0.853	0.828 (0.804–0.851)	0.737	0.758	0.737	0.735	0.736	0.782
Support vector machine	0.829	0.737 (0.710–0.764)	0.585	0.829	0.585	0.712	0.642	0.715
k-Nearest neighbor	0.828	0.670 (0.642–0.699)	0.703	0.552	0.703	0.563	0.625	0.376
Decision tree	0.768	0.616 (0.587–0.645)	0.382	0.849	0.382	0.582	0.461	0.444
Random forest	0.839	0.820 (0.796–0.843)	0.701	0.777	0.701	0.782	0.739	0.808
XGBoost	0.857	0.837 (0.814–0.860)	0.755	0.801	0.755	0.825	0.788	0.855

Abbreviations: AUC: area under the receiver operating characteristic curve, CI: confidence interval, XGBoost: Extreme Gradient Boosting, MCC: matthews correlation coefficient.

### Model explainability

**Figure 3.**
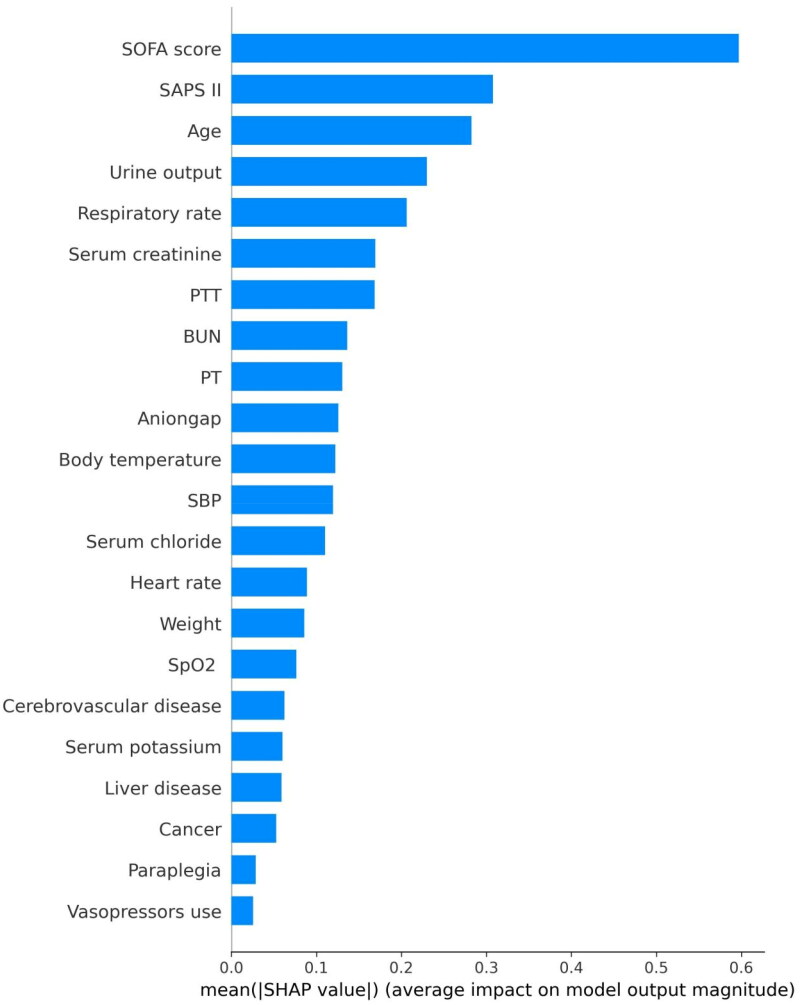
The important features derived from the XGBoost model. Ranking of feature importance indicated by SHAP. The matrix plot depicts the importance of each covariate in the development of the final predictive model. Abbreviations: SHAP: SHapley Additive explanation, SOFA: sequential organ failure assessment, SAPS II: simplified acute physiology score II, PTT: partial thromboplastin time, BUN: blood urea nitrogen, PT: prothrombin time, SpO_2_: oxygen saturation, MAP: mean arterial pressure.

**Figure 4.**
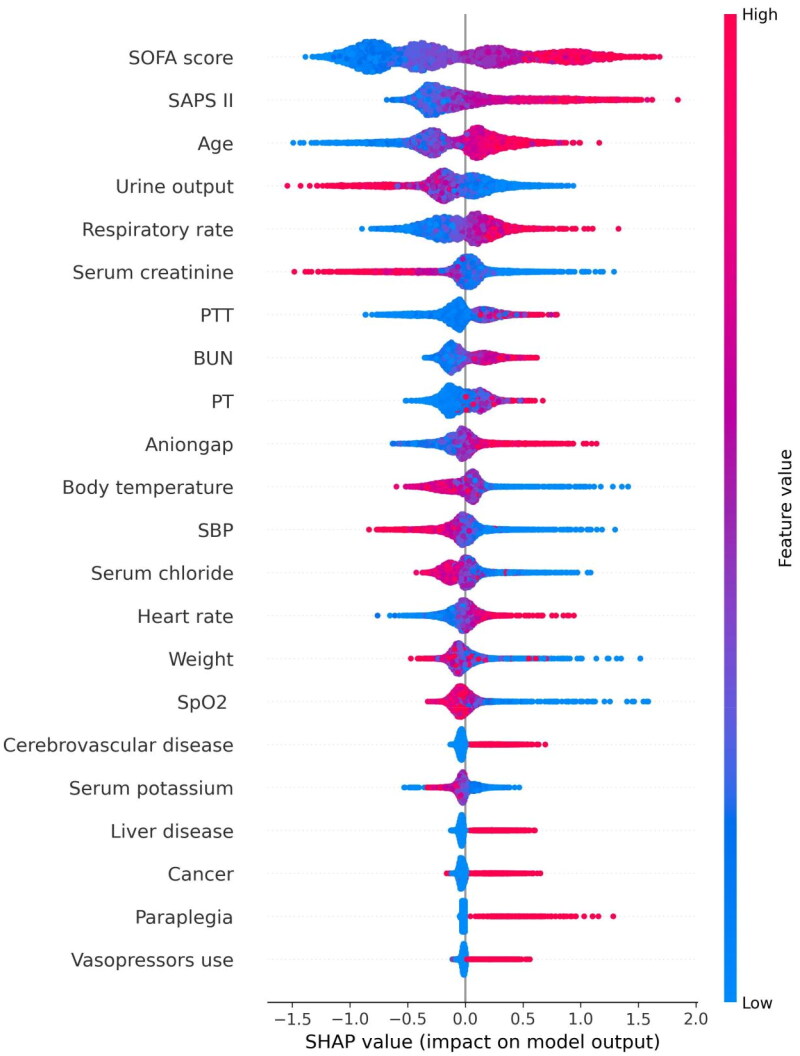
SHAP summary plot of the features of the XGBoost model. The higher the SHAP value of a feature, the higher the probability of death development. Each line represents a feature, and the abscissa is the SHAP value. Red dots represent higher feature values, and blue dots represent lower feature values. Abbreviations: SHAP: SHapley Additive explanation, SOFA: sequential organ failure assessment, SAPS II: simplified acute physiology score II, PTT: partial thromboplastin time, BUN: blood urea nitrogen, PT: prothrombin time, SpO_2_: oxygen saturation, MAP: mean arterial pressure.

**Figure 5.**
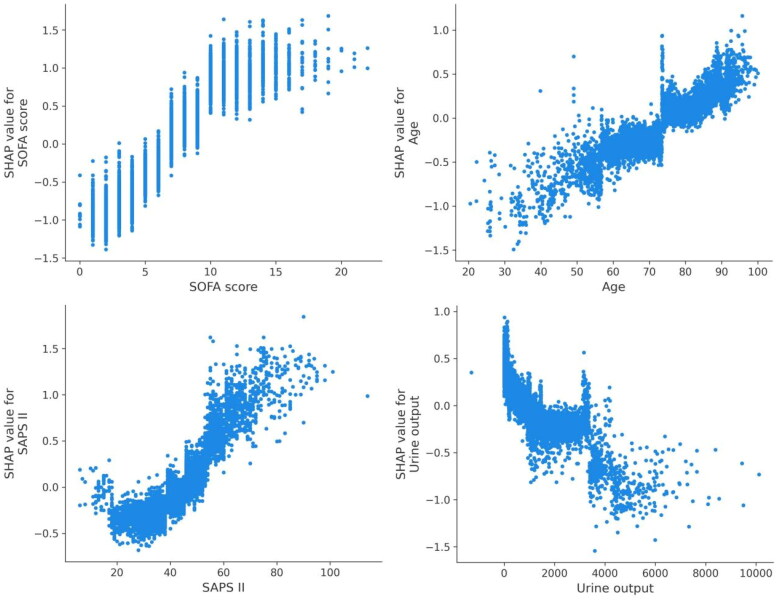
SHAP dependence plot of the XGBoost model. SHAP values for specific features exceed zero, representing an increased risk of death development. Abbreviations: SHAP: Shapley Additive explanation, SOFA: sequential organ failure assessment, SAPS II: simplified acute physiology score II.

Utilizing SHAP values, our goal was to elucidate the mortality prediction mechanism employed by the XGBoost model. Figure 3 illustrates the feature importance ranking of the XGBoost model through SHAP summary plots, highlighting SOFA score, age, SAPS II, and urine output as the four primary contributors to the model. To provide more detailed information about Figure 3, we provide dependence plots of the top four most weighted clinical features output by the XGBoost prediction model to show the relationship between the feature values and the SHAP values of the features (Figures 4 and 5).

**Figure 6.**
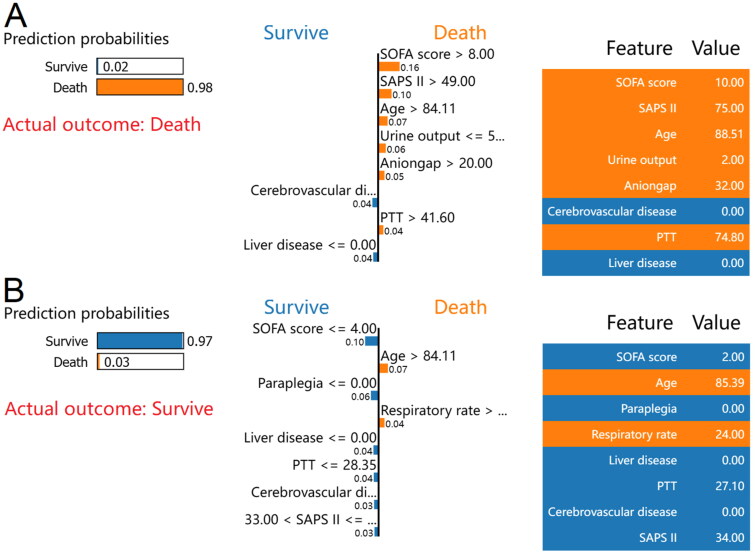
LIME algorithm for explaining individual’s prediction results. Screenshot of the death prognosis for critically ill patients with CHF combined with CKD. (A) Utilizing the LIME method, show a death case. (B) Present a case of survival using the LIME method. The left portion of the picture depicts expected LIME findings. The center section lists, from highest to lowest, the eight variables that had the greatest impact on survival or death. The length of the bar for each feature reflects the weight of that feature in the prediction. A longer bar represents a characteristic that contributes more to survival or mortality. The right panel displays the crucial values of these eight factors at which they had the greatest influence on survival or death. Abbreviations: LIME: Local Interpretable Model-Agnostic Explanations, CHF: congestive heart failure, CKD: chronic kidney disease, SOFA: sequential organ failure assessment, SAPS II: simplified acute physiology score II, PTT: partial thromboplastin time.

The LIME method was then used for two random samples from the validation set to provide insight into the individual mortality forecast. The case of death, as reported by the LIME algorithm, is depicted in Figure 6(A). According to the XGBoost model, 98% was the estimated probability of death. A SOFA score of 10, SAPS II of 75, age of 88.51 years, urine volume of 2 mL, anion gap of 32 mEq/L, and PTT of 74.8 s were all associated with an increased risk of death in the XGBoost model. The lack of a history of cerebrovascular disease or liver disease was discovered to lessen the probability of mortality. For this case, both the XGBoost model and the actual outcome were death. Similarly, Figure 6(B) depicts a survival case with the LIME method. According to the XGBoost model, the probability of mortality was 3%. An age of 85.39 years, and a respiratory rate of 24 breaths/min in the XGBoost model was associated with an increased risk of death. In contrast, a SOFA score of 2, SAPS II of 34, PTT of 27.1 s, and the absence of paraplegia, liver disease, or cerebrovascular disease reduced the risk of death. The XGBoost model for this patient predicted survival, and survival was also the actual outcome.

### Subgroup analyses

Subgroup analyses for the presence or absence of sepsis, diabetes, paraplegia, cancer, AKI and different CKD stages showed that the sustained robustness of the XGBoost model in predicting mortality among these patients. Comprehensive results can be found in Supplementary Figure S3.

## Discussion

This study involved the development and validation of six models, incorporating 22 clinical factors, to predict in-hospital mortality among critically ill patients with CHF combined with CKD. Notably, the XGBoost model surpassed other models (KNN, SVM, RF, decision tree and logistic regression) as well as traditional risk scores (SAPS II, SOFA score and GWTG-HF) in predicting death in critically ill patients with CHF combined with CKD. Analysis of feature importance revealed that the SOFA score, age, SAPS II, and urine volume constituted the top four features with the most significant impact on the XGBoost model’s prediction of in-hospital mortality. In addition, we describe how these factors influence the XGBoost model. These insights contribute to a comprehensive understanding of ML models for predicting in-hospital mortality in critically ill patients with CHF combined with CKD.

More than one million primary and roughly three million secondary hospital admissions occur annually in the United States due to heart failure (HF), a condition linked with a high mortality risk and significant morbidity [13–15]. Thus, it significantly burdens impacted people and global healthcare systems. HF frequently coexists with various prognosis-relevant comorbidities and directly affects other organs, such as the kidneys. The progression of HF or kidney illness might negatively impact patient outcomes by activating vicious cycles that frequently accelerate cardiac and renal deterioration [16,17]. A study conducted in the United States discovered that hospitalization rates for HF were high among patients with CKD and that individuals with CKD combined with HF had an increased risk of CKD progression and death [18]. To mitigate mortality, it is necessary to establish and advocate for predictive models that can precisely and promptly identify patients at a heightened risk of clinical deterioration.

In our comparative analysis, the p-value for the difference in AUC between the XGBoost and Logistic regression models was not statistically significant. However, it is crucial to note that the selection of an optimal model goes beyond statistical significance. The practical effectiveness of XGBoost in predicting in-hospital mortality for critically ill patients with congestive heart failure combined with chronic kidney disease is evident in several aspects. XGBoost excels in capturing complex, nonlinear relationships within the dataset, a vital consideration given the intricate nature of critically ill patients. Additionally, the model’s interpretability is enhanced through the use of SHAP values and the LIME algorithm, providing insights into influential factors. The robustness of XGBoost across diverse datasets and its potential for better generalization further contribute to its practical superiority. Moreover, a comprehensive evaluation considering metrics beyond AUC, such as sensitivity, specificity, and positive predictive value, consistently demonstrates the favorable performance of XGBoost. Despite the lack of statistical significance in the AUC comparison, the nuanced strengths of XGBoost collectively support its effectiveness in predicting in-hospital mortality, underscoring the significance of our findings in a clinical context.

In this investigation, the XGBoost model demonstrated superior predictive accuracy for in-hospital mortality in critically ill patients with CHF combined with CKD compared to other models. These findings align with numerous other studies in the field. Li et al. demonstrated that the XGBoost model surpassed other models, including SVM, RF, and logistic regression in predicting in-hospital mortality among ICU patients with HF [19]. Hu et al. found that XGBoost outperformed RF, naive bayes, decision trees, logistic regression, KNN, and SVM in predicting in-hospital mortality among critically ill patients with acute kidney injury [20]. As per a meta-analysis, XGBoost demonstrated superior performance in predicting acute kidney injury compared to other ML techniques, including bayesian networks and SVM [21]. Moreover, conventional severity scoring methods, including the SAPS II, SOFA score and GWTG-HF, exhibited subpar performance compared to ML models. This suggests that traditional scoring tools may not be reliable for predicting mortality in critically ill patients with CHF combined with CKD.While the SAPS II, SOFA score and GWTG-HF are capable of estimating the likelihood of adverse outcomes in critically ill patients, their exclusion of a significant number of pertinent parameters in their studies may lead to less accurate predictions compared to multivariable models [22]. Prior research has indicated that the SAPS II, SOFA score and GWTG-HF have inferior prediction ability compared to ML models [6].

In this study, the ML algorithm was used for the first time to predict in-hospital mortality in critically ill patients with CHF combined with CKD. In critically ill patients with CHF combined with CKD, a complicated XGBoost model revealed that SOFA score, age, SAPS II, and urine output were most strongly linked with mortality. The SOFA score is a tool that describes the presence of organ dysfunction [23]. It assigns a daily score between 1 and 4 to each of the six organ systems based on the severity of dysfunction: respiratory, circulatory, renal, hematologic, hepatic, and central nervous systems [24]. The association between SOFA scores and clinical outcomes was high [25]. Similarly, in the present investigation, the SOFA score had the maximum weight in the XGBoost model. It was determined to be the most significant predictor of mortality in critically ill patients with CHF combined with CKD. Age is a significant risk factor for mortality in critically ill patients with CHF combined with CKD. Numerous studies have demonstrated that aging is associated with an increased risk of death in critically ill patients with CHF combined with CKD. In our investigation, the median age of non-survivors was older than the median age of survivors. SAPS II is also a significant predictor of mortality. The SAPS II includes seventeen variables, and higher total scores are indicative of greater illness severity [26]. Prior studies have established an association between SAPS II and an elevated mortality rate among ICU patients [27]. In addition, we discovered a correlation between urine output and mortality in critically ill patients with CHF combined with CKD. Oliguria is a prevalent condition among ICU patients and represents the primary cause of renal parenchymal damage [28]. Numerous studies have illustrated a correlation between reduced urine output and unfavorable outcomes in critically ill individuals [28,29].

However, this study also has some limitations. First, due to its retrospective design, this can lead to unavoidable selection bias. Second, different comorbidities may somewhat mask outcomes in patients with CKD and CHF. Third, the current study is a single-center study, and the results may not be extrapolated to other centers. In addition, prospective and multicenter studies are needed to validate this study’s findings further.

## Conclusions

In conclusion, ML models emerge as dependable tool for mortality prediction in critically ill patients with CHF combined with CKD. Among all the prediction models, the XGBoost model stands out as the most effective, offering clinicians a valuable tool for accurately management and timely interventions to mitigate mortality risks in critically ill patients with CHF combined with CKD who are at high risk of death.

Notably, among all the predictive models, the XGBoost model stands out as the most effective, offering clinicians a valuable tool for accurate management and timely interventions to mitigate mortality risks in critically ill patients with CHF combined with CKD who are at elevated risk of death."

## Supplementary Material

Supplemental Material

## Data Availability

The datasets presented in the current study are available in the MIMIC IV database (https://physionet.org/content/mimiciv/1.0/).

## References

[1] Cleland JG, Khand A, Clark A. The heart failure epidemic: exactly how big is it? Eur Heart J. 2001;22(8):1–12. Epub 2001/04/05. doi: 10.1053/euhj.2000.2493. 11286518

[2] McAlister FA, Ezekowitz J, Tonelli M, et al. Renal insufficiency and heart failure: prognostic and therapeutic implications from a prospective cohort study. Circulation. 2004;109(8):1004–1009. Epub 2004/02/11. doi: 10.1161/01.cir.0000116764.53225.a9. 14769700

[3] Damman K, Valente MA, Voors AA, et al. Renal impairment, worsening renal function, and outcome in patients with heart failure: an updated meta-analysis. Eur Heart J. 2014;35(7):455–469. Epub 2013/10/30. doi: 10.1093/eurheartj/eht386. 24164864

[4] Damman K, Testani JM. The kidney in heart failure: an update. Eur Heart J. 2015;36(23):1437–1444. Epub 2015/04/04. doi: 10.1093/eurheartj/ehv010. 25838436 PMC4465636

[5] Alam N, Hobbelink EL, van Tienhoven AJ, et al. The impact of the use of the early warning score (ews) on patient outcomes: a systematic review. Resuscitation. 2014;85(5):587–594. Epub 2014/01/29. doi: 10.1016/j.resuscitation.2014.01.013. 24467882

[6] Hou N, Li M, He L, et al. Predicting 30-Days mortality for mimic-Iii patients with sepsis-3: a machine learning approach using xgboost. J Transl Med. 2020;18(1):462. doi: 10.1186/s12967-020-02620-5. 33287854 PMC7720497

[7] Du M, Haag DG, Lynch JW, et al. Comparison of the tree-based machine learning algorithms to cox regression in predicting the survival of oral and pharyngeal cancers: analyses based on seer database. Cancers (Basel). 2020;12(10):2802. Epub 2020/10/03. doi: 10.3390/cancers12102802. 33003533 PMC7600270

[8] Weis C, Cuénod A, Rieck B, et al. Direct antimicrobial resistance prediction from clinical Maldi-Tof mass spectra using machine learning. Nat Med. 2022;28(1):164–174. doi: 10.1038/s41591-021-01619-9. 35013613

[9] Hu C, Li L, Huang W, et al. Interpretable machine learning for early prediction of prognosis in sepsis: a discovery and validation study. Infect Dis Ther. 2022;11(3):1117–1132. Epub 2022/04/12. doi: 10.1007/s40121-022-00628-6. 35399146 PMC9124279

[10] Zhang Z, Chen L, Xu P, et al. Effectiveness of automated alerting system compared to usual care for the management of sepsis. NPJ Digit Med. 2022;5(1):101. doi: 10.1038/s41746-022-00650-5. 35854120 PMC9296632

[11] Zhou S, Zeng Z, Wei H, et al. Early combination of albumin with crystalloids administration might be beneficial for the survival of septic patients: a retrospective analysis from mimic-Iv database. Ann Intensive Care. 2021;11(1):42. Epub 2021/03/11. doi: 10.1186/s13613-021-00830-8. 33689042 PMC7947075

[12] Johnson AE, Pollard TJ, Shen L, et al. Mimic-Iii, a freely accessible critical care database. Sci Data. 2016;3:160035. doi: 10.1038/sdata.2016.35. 27219127 PMC4878278

[13] Schocken DD, Benjamin EJ, Fonarow GC, et al. Prevention of heart failure: a scientific statement from the American heart association councils on epidemiology and prevention, clinical cardiology, cardiovascular nursing, and high blood pressure research; quality of care and outcomes research interdisciplinary working group; and functional genomics and translational biology interdisciplinary working group. Circulation. 2008;117(19):2544–2565. Epub 2008/04/09. doi: 10.1161/circulationaha.107.188965. 18391114

[14] van Riet EE, Hoes AW, Wagenaar KP, et al. Epidemiology of heart failure: the prevalence of heart failure and ventricular dysfunction in older adults over time. A systematic review. Eur J Heart Fail. 2016;18(3):242–252. Epub 2016/01/05. doi: 10.1002/ejhf.483. 26727047

[15] Redfield MM, Jacobsen SJ, Burnett JC, Jr., et al. Burden of systolic and diastolic ventricular dysfunction in the community: appreciating the scope of the heart failure epidemic. Jama. 2003;289(2):194–202. Epub 2003/01/09. doi: 10.1001/jama.289.2.194. 12517230

[16] Bagshaw SM, Cruz DN, Aspromonte N, et al. Epidemiology of Cardio-Renal syndromes: workgroup statements from the 7th adqi consensus conference. Nephrol Dial Transplant. 2010;25(5):1406–1416. Epub 2010/02/27. doi: 10.1093/ndt/gfq066. 20185818

[17] House AA, Anand I, Bellomo R, et al. Definition and classification of Cardio-Renal syndromes: workgroup statements from the 7th adqi consensus conference. Nephrol Dial Transplant. 2010;25(5):1416–1420. Epub 2010/03/17. doi: 10.1093/ndt/gfq136. 20228069

[18] Bansal N, Zelnick L, Bhat Z, et al. Burden and outcomes of heart failure hospitalizations in adults with chronic kidney disease. J Am Coll Cardiol. 2019;73(21):2691–2700. Epub 2019/05/31. doi: 10.1016/j.jacc.2019.02.071. 31146814 PMC6590908

[19] Li J, Liu S, Hu Y, et al. Predicting mortality in intensive care unit patients with heart failure using an interpretable machine learning model: retrospective cohort study. J Med Internet Res. 2022;24(8):e38082. doi: 10.2196/38082. 35943767 PMC9399880

[20] Hu C, Tan Q, Zhang Q, et al. Application of interpretable machine learning for early prediction of prognosis in acute kidney injury. Comput Struct Biotechnol J. 2022;20:2861–2870. Epub 2022/06/30. doi: 10.1016/j.csbj.2022.06.003. 35765651 PMC9193404

[21] Song X, Liu X, Liu F, et al. Comparison of machine learning and logistic regression models in predicting acute kidney injury: a systematic review and Meta-Analysis. Int J Med Inform. 2021;151:104484. Epub 2021/05/16. doi: 10.1016/j.ijmedinf.2021.104484. 33991886

[22] Wu J, Huang L, He H, et al. Red cell distribution width to platelet ratio is associated with increasing in-hospital mortality in critically ill patients with acute kidney ­injury. Dis Markers. 2022;2022:4802702. doi: 10.1155/2022/4802702. 35082929 PMC8786548

[23] He Y, Xu J, Shang X, et al. Clinical characteristics and risk factors associated with icu-Acquired infections in sepsis: a retrospective cohort study. Front Cell Infect Microbiol. 2022;12:962470. Epub 2022/08/16. doi: 10.3389/fcimb.2022.962470. 35967847 PMC9366915

[24] Minne L, Abu-Hanna A, de Jonge E. Evaluation of sofa-based models for predicting mortality in the icu: a systematic review. Crit Care. 2008;12(6):R161–Epub 2008/12/19. doi: 10.1186/cc7160. 19091120 PMC2646326

[25] Zhu Y, Zhang R, Ye X, et al. Saps iii is superior to sofa for predicting 28-Day mortality in sepsis patients based on sepsis 3.0 criteria. Int J Infect Dis. 2022;114:135–141. Epub 2021/11/15. doi: 10.1016/j.ijid.2021.11.015. 34775116

[26] Le Gall JR, Lemeshow S, Saulnier F. A new simplified acute physiology score (saps Ii) based on a european/North American multicenter study. Jama. 1993;270(24):2957–2963. Epub 1993/12/22. doi: 10.1001/jama.270.24.2957. 8254858

[27] Mirzakhani F, Sadoughi F, Hatami M, et al. Which model is superior in predicting icu survival: artificial intelligence versus conventional approaches. BMC Med Inform Decis Mak. 2022;22(1):167. Epub 2022/06/28. doi: 10.1186/s12911-022-01903-9. 35761275 PMC9235201

[28] Macedo E, Malhotra R, Bouchard J, et al. Oliguria is an early predictor of higher mortality in critically ill patients. Kidney Int. 2011;80(7):760–767. Epub 2011/07/01. doi: 10.1038/ki.2011.150. 21716258

[29] Kellum JA, Sileanu FE, Murugan R, et al. Classifying aki by urine output versus serum creatinine level. J Am Soc Nephrol. 2015;26(9):2231–2238. Epub 2015/01/09. doi: 10.1681/asn.2014070724. 25568178 PMC4552117

