# Supplementary material for: Machine learning algorithm for predict the in-hospital mortality in critically ill patients with congestive heart failure combined with chronic kidney disease
Source: Ren Fail. 2024 Feb 15;46(1):2315298. doi: 10.1080/0886022X.2024.2315298 (PMC10877653; doi:10.1080/0886022X.2024.2315298)
Supplement: Supplemental Material [file IRNF_A_2315298_SM3664.pdf]

Table S1. ICD codes for CHF and CKD

Disease	ICD-9	ICD-10
CHF	39891, 40201, 40211, 40291, 40401, 40403, 40411, 40413, 40491, 40493, 4254, 4255, 4257, 4258, 4259, 4280, 4281, 42820, 42821, 42822, 42823, 42830, 42831, 42832, 42833, 42840, 42841, 42842, 42843, 4289	I509, I099, I110, I130, I132, I255, I420, I425, I426, I427, I428, I429, I43, I50, I501, I502, I5020, I5021, I5022, I5023, I503, I5030, I5031, I5032, I5033, I504, I5040, I5041, I5042, I5043, I508, I5081, I50810, I50811, I50812, I50813, I50814, I5082, I5083, I5084, I5089, P290
CKD	5851, 5852, 5853, 5854, 5855, 5856, 5859	N18, N181, N182, N183, N184, N185, N186, N189

Abbreviations: ICD: International Classification of Diseases, CHF: congestive heart failure, CKD: chronic kidney disease.

Table S2. Missing number (%) for variables

Variables	Missing number (%)
**Demographic features**	
Sex	0(0)
Age	0(0)
Weight	258(5.1%)
Ethnicity, n (%)	0(0)
**CKD stage**	0(0)
**Comorbidities**	
Myocardial infarction	0(0)
Peripheral vascular disease	0(0)
Cerebrovascular disease	0(0)
Dementia	0(0)
COPD	0(0)
Rheumatic disease	0(0)
Peptic ulcer disease	0(0)
Liver disease	0(0)
Diabetes	0(0)
Paraplegia	0(0)
Cancer	0(0)
Aids	0(0)
Sepsis	0(0)
AKI	0(0)
**Vital signs**	
Heart rate	2(0.0%)
MAP	9(0.2%)

SBP	19(0.4%)
Respiratory rate	13(0.3%)
Body temperature	32(0.6%)
SpO_2	1(0.0%)
**Biochemical indices**	
Hematocrit	10(0.2%)
Hemoglobin	10(0.2%)
Platelets	10(0.2%)
WBC	10(0.2%)
Anion gap	6(0.1%)
Bicarbonate	6(0.1%)
BUN	6(0.1%)
Serum calcium	66(1.3%)
Serum chloride	5(0.1%)
Serum creatinine	6(0.1%)
Serum glucose	9(0.2%)
Serum sodium	5(0.1%)
Serum potassium	5(0.1%)
INR	176(3.5%)
PT	176(3.5%)
PTT	189(3.7%)
**Urine output**	408(8.1%)
**Treatments**	
RRT	0(0)
Vasopressors use	0(0)
Mechanical ventilation	0(0)

**Severity scores of illness**	
SOFA score	0(0)
SAPS II	0(0)

Abbreviations: CKD: chronic kidney disease, Aids: acquired immune deficiency syndrome, AKI: acute kidney injury, COPD: chronic obstructive pulmonary disease, MAP: mean arterial pressure, SBP: systolic blood pressure, SpO_2: oxygen saturation, WBC: white blood cell, BUN: blood urea nitrogen, INR: international normalized ratio, PT: prothrombin time, PTT: partial thromboplastin time, RRT: renal replacement therapy, SOFA: sequential organ failure assessment, SAPS II: simplified acute physiology score II.

Table S3. Clinical features used for developing the models.

Variables
**Demographics**
Age
Weight
**Comorbidities**
Cerebrovascular disease
Liver disease
Paraplegia
Cancer
**Vital signs**
Heart rate
SBP
Respiratory rate
Body temperature
SpO_2
**Biochemical indices**
Anion gap
BUN
Serum chloride
Serum creatinine
Serum potassium
PT
PTT

Urine output

Medical treatments

Vasopressors use

Severity scores of illness

SOFA score

SAPS II

Abbreviations: SBP: systolic blood pressure, SpO_2: oxygen saturation, BUN: blood urea nitrogen, PT: prothrombin time, PTT: partial thromboplastin time, SOFA: sequential organ failure assessment, SAPS II: simplified acute physiology score II.

Table S4. Correlation coefficients of the clinical features.

	Age	Weight	Cerebrovascular disease	Liver disease	Paraplegia	Cancer	Heart rate	Respiratory rate	Body temperature	SpO_2	Anion gap	BUN	Serum chloride	Serum creatinine	Serum potassium	PT	PTT	Vasopressors use	Urine output	SOFA score	SAPS II	SBP
Age	1																					
Weight	-0.25	1																				
Cerebrovascular disease	0.03	-0.05	1																			
Liver disease	-0.13	0.04	-0.04	1																		
Paraplegia	0.03	-0.03	0.32	-0.02	1																	
Cancer	0.07	-0.04	-0.04	0.03	0	1																
Heart rate	-0.1	0.01	-0.01	0.03	0	0.08	1															
Respiratory rate	0.03	-0.01	-0.01	0.01	-0.01	0.07	0.31	1														
Body	-0.11	0.06	0.01	-0.01	0.01	0.04	0.22	0.14	1													

temperature																					
SpO_2	-0.02	-0.05	0.03	0	0.02	-0.05	-0.12	-0.15	-0.03	1											
Anion gap	-0.13	-0.05	0	0.05	-0.02	-0.02	0.1	0.12	-0.03	-0.07	1										
BUN	-0.02	0.03	-0.05	0.05	-0.04	-0.01	-0.05	0.02	-0.11	-0.03	0.37	1									
Serum chloride	0.15	-0.06	0.05	-0.02	0.04	0.02	-0.02	-0.04	-0.04	0.07	-0.35	-0.17	1								
Serum creatinine	-0.32	-0.01	-0.02	0.03	-0.02	-0.05	0	-0.01	-0.03	0	0.48	0.43	-0.25	1							
Serum potassium	-0.07	0.01	-0.02	0.01	-0.01	-0.02	-0.03	0.04	-0.05	-0.01	0.28	0.24	-0.09	0.3	1						
PT	0.05	0.03	-0.04	0.03	-0.03	-0.01	0.03	0.03	-0.06	-0.04	0.12	0.12	-0.08	0.01	0.02	1					
PTT	0.04	0	0.01	0.03	-0.01	-0.02	-0.02	-0.01	-0.09	-0.01	0.04	0	0	-0.02	-0.04	0.18	1				
Vasopressors use	-0.02	0.02	-0.01	0.03	-0.01	-0.01	0.09	0.01	-0.05	-0.06	0.1	0.01	0.02	0.02	-0.01	0.06	0.06	1			
Urine output	-0.06	0.19	-0.04	-0.03	-0.02	-0.01	-0.04	0.03	0.04	-0.01	-0.13	0.03	0.01	-0.24	-0.05	0.01	-0.01	-0.12	1		
SOFA score	-0.07	0	0.04	0.16	0.01	0	0.08	0.03	-0.08	-0.03	0.22	0.16	-0.01	0.23	0.1	0.08	0.08	0.4	-0.29	1	
SAPS II	0.32	-0.09	0.03	0.06	0.01	0.18	0.08	0.07	-0.11	-0.05	0.15	0.21	0.07	0.05	0.13	0.08	0.07	0.3	-0.35	0.63	1

SBP	-0.08	-0.04	0.06	-0.05	0.04	-0.02	-0.01	0.06	0.07	0.02	0	-0.02	0.05	0.08	0.05	-0.11	-0.08	-0.16	0.08	-0.23	-0.23	1

Abbreviations: SBP: systolic blood pressure, SpO_2: oxygen saturation, BUN: blood urea nitrogen, PT: prothrombin time, PTT: partial thromboplastin time, SOFA: sequential organ failure assessment, SAPS II: simplified acute physiology score II.

Table S5. Comparison of baseline characteristics between the training and test sets

Variables	Total (n =5041)	Training set (n =3528)	Test set (n =1513)	*P* value
**Demographics**				
Age (years)	76.9 [67.9, 84.8]	76.6 [67.8, 84.7]	77.6 [68.0, 84.9]	0.417
Weight (kg)	79.7 [67.0, 94.5]	79.4 [67.0, 94.5]	80.0 [67.3, 94.6]	0.511
**Comorbidities, n (%)**				
Cerebrovascular disease	661 (13.1)	463 (13.1)	198 (13.1)	1.000
Liver disease	514 (10.2)	364 (10.3)	150 (9.9)	0.702
Paraplegia	149 (3.0)	103 (2.9)	46 (3.0)	0.888
Cancer	570 (11.3)	396 (11.2)	174 (11.5)	0.814
**Vital signs**				
Heart rate (beats/minute)	84.0 [72.0, 97.0]	84.0 [73.0, 97.0]	83.0 [71.0, 98.0]	0.232
SBP (mmHg)	120.0 [104.0, 140.0]	120.0 [104.0, 140.0]	121.0 [104.0, 139.8]	0.942
Respiratory rate (beats/minute)	19.0 [16.0, 23.0]	19.0 [16.0, 23.0]	19.0 [16.0, 23.0]	0.550
Body temperature (°C)	36.6 [36.3, 36.9]	36.6 [36.4, 36.9]	36.7 [36.3, 36.9]	0.365
SpO_2 (%)	98 [95, 100]	98 [95, 100]	98 [95, 100]	0.532
**Biochemical indices**				
Anion gap (mEq/L)	16.0 [14.0, 19.0]	16.0 [14.0, 19.0]	16.0 [14.0, 19.0]	0.511
BUN (mg/dL)	41.0 [28.0, 62.0]	41.0 [27.0, 62.0]	42.0 [28.0, 63.0]	0.473
Serum chloride (mEq/l)	101 [96, 106]	101 [97, 106]	102 [96, 106]	0.147
Serum creatinine (mg/dL)	2.00 [1.40, 3.20]	2.00 [1.40, 3.20]	2.00 [1.50, 3.30]	0.441
Serum potassium (mEq/L)	4.40 [4.00, 5.00]	4.50 [4.00, 5.00]	4.40 [4.00, 5.00]	0.985
PT (s)	14.7 [12.7, 19.4]	14.7 [12.7, 19.5]	14.7 [12.7, 19.3]	0.626
PTT (s)	32.6 [28.2, 41.1]	32.70 [28.3, 41.3]	32.3 [28.1, 40.9]	0.409

**Urine output (mL)**	1270 [635, 2145]	1265 [630, 2140]	1281 [650, 2160]	0.843
**Medical treatments, n (%)**				
Vasopressors use	307 (6.1)	207 (5.9)	100 (6.6)	0.344
**Severity scores of illness**				
SOFA score	6.00 [4.00, 9.00]	6.00 [4.00, 9.00]	6.00 [4.00, 9.00]	0.032
SAPS II	42.0 [35.0, 51.0]	42.0 [35.0, 50.0]	42.0 [35.0, 51.0]	0.077

Abbreviations: SBP: systolic blood pressure, SpO_2: oxygen saturation, BUN: blood urea nitrogen, PT: prothrombin time, PTT: partial thromboplastin time, SOFA: sequential organ failure assessment, SAPS II: simplified acute physiology score II.

Table S6. Comparison of the AUCs between the six models using the DeLong method

	Logistic regression	SVM	KNN	Decision tree	Random forest	XGBoost
Logistic regression						
SVM	<0.001					
KNN	<0.001	<0.001				
Decision tree	<0.001	<0.001	<0.001			
Random forest	0.496	<0.001	<0.001	<0.001		
XGBoost	0.425	<0.001	<0.001	<0.001	<0.001	

Abbreviations: AUC: area under the receiver operating characteristic curve, CI: confidence interval, SVM: support vector machine, KNN, k-nearest neighbors, XGBoost: Extreme Gradient Boosting.

Table S7. AUC of the six models in the training set.

**Models**	**AUC (95% CI)**
Logistic regression	0.819 (0.803–0.835)
Support vector machine	0.753 (0.736–0.771)
k-Nearest neighbor	0.891 (0.878–0.904)
Decision tree	1.000 (1.000–1.000)
Random forest	1.000 (1.000–1.000)
XGBoost	0.984 (0.979–0.989)

Abbreviations: AUC: area under the receiver operating characteristic curve, CI: confidence interval, XGBoost: Extreme Gradient Boosting.

Figure S1. Feature selection using the LASSO regression model

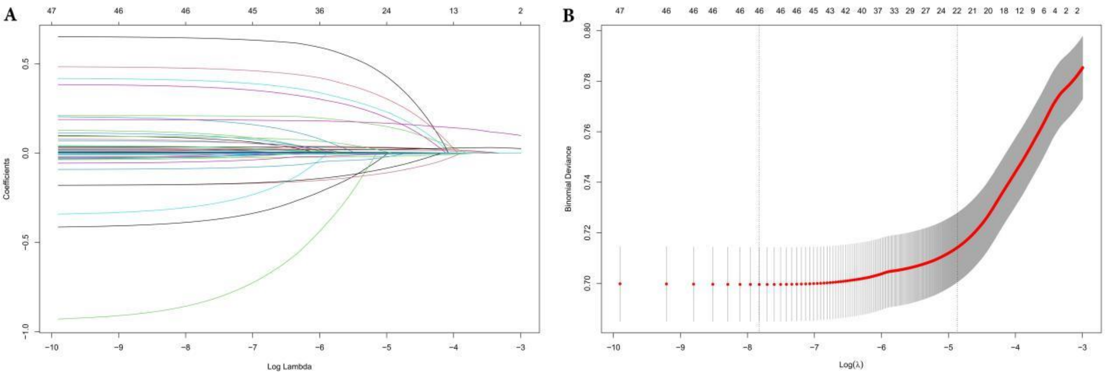

(A) LASSO coefficient profiles of the 47 baseline features.

(B) Tuning parameter (λ) selection in the LASSO model used 10-fold cross-validation via minimum criteria.

Abbreviations: LASSO: least absolute shrinkage and selection operator.

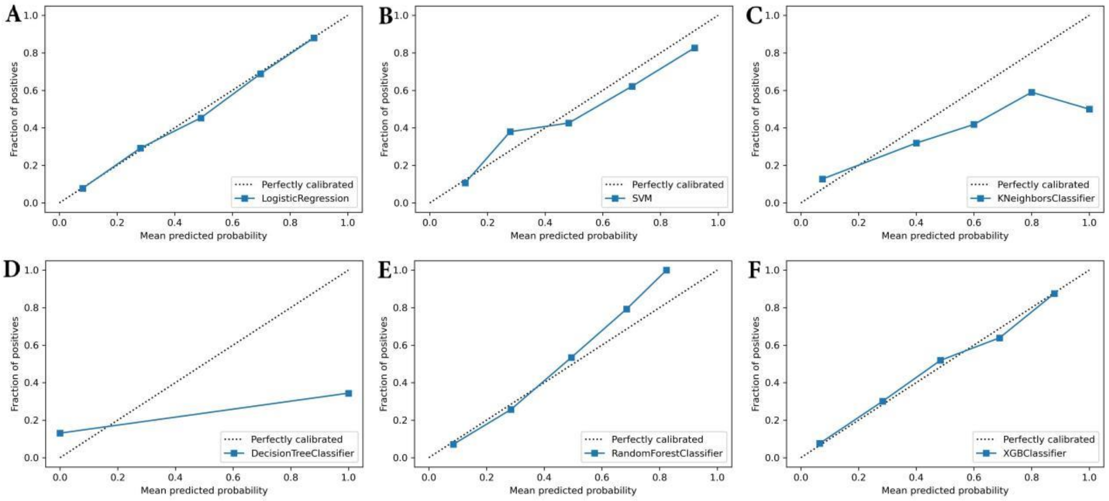

Figure S2. The calibration curves for the six models

Abbreviations: SVM: support vector machine.

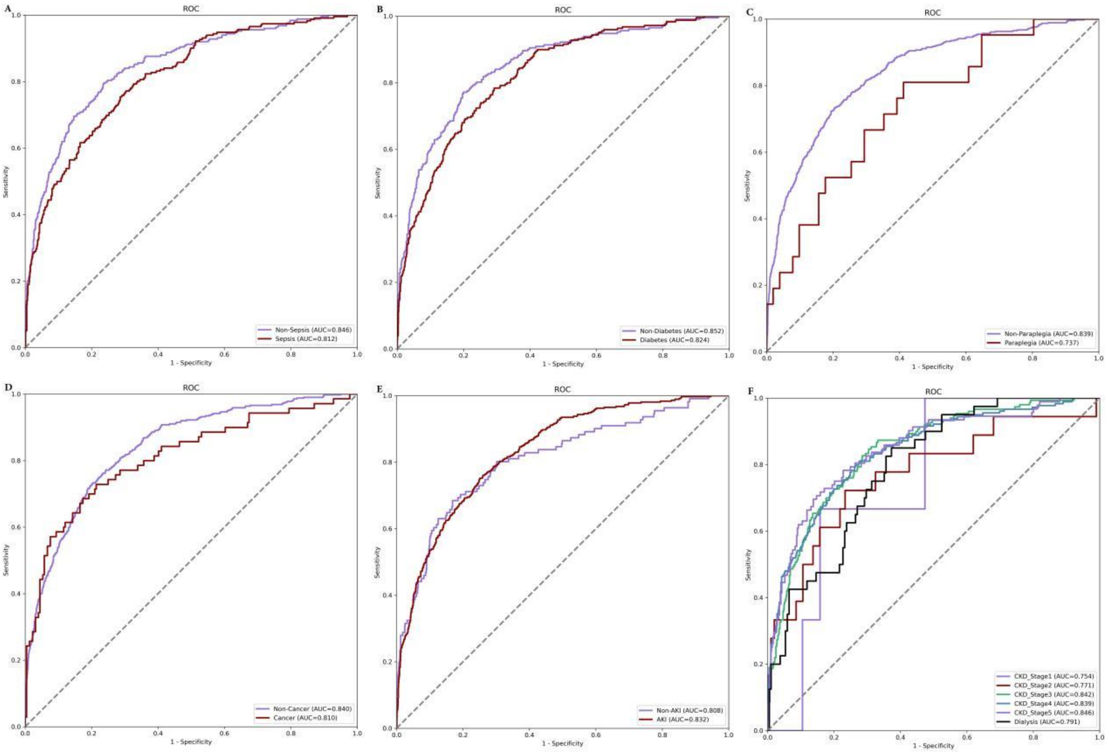

Figure S3. ROC curves of the XGBoost model for predicting in-hospital mortality in patients with CKD combined with CHF in different subgroups

Abbreviations: SVM: support vector machine. ROC: receiver operating characteristic, CKD: chronic kidney disease, CHF: congestive heart failure, AKI: acute kidney injury.
